# Lung function and plasma biomarkers: Unraveling the interplay with Alzheimer’s Disease and Related Dementias

**DOI:** 10.1002/alz.088227

**Published:** 2025-01-03

**Authors:** Sithara Vivek, Eileen M. Crimmins, Weihua Guan, Jessica D Faul, Bharat Thyagarajan

**Affiliations:** ^1^ University of Minnesota, Minneapolis, MN USA; ^2^ University of Southern California, Los Angeles, CA USA; ^3^ University of Michigan, Ann Arbor, MI USA

## Abstract

**Background:**

With the emerging role of the blood biomarkers in Alzheimer’s Disease (AD) clinical practice and trials, it is crucial to identify and address factors influencing the concentrations of these biomarkers in circulation for enhanced clinical utility. We aim to assess the impact of lung function on plasma AD biomarker levels and elucidate the relationship between lung function and Alzheimer’s Disease and Related Dementias (ADRD).

**Method:**

We used the Peak Expiratory Flow (PEF), plasma biomarkers of AD (Amyloid beta 42/40 (Aβ42/40) ratio, phosphorylated‐tau181 (p‐tau181), Neurofilament light chain (NfL) and Glial fibrillary acidic protein (GFAP)) measured in the Health and Retirement Study (HRS) 2016 survey participants (n = 3801) and incident dementia (n = 142) over 4 years. Participants were categorized into sex‐specific standardized residual percentiles (SRP) of PEF adjusted for age and height based on previously published method. We created 3 categories using SRP with cut points at: ≥50%, 10%–50%, and <10% (poor lung function). Multiple linear regressions assessed the association between PEF and AD biomarkers, adjusting for sample weights, age, race, education, body mass index, smoking status, comorbidity index, composite inflammatory score, estimated glomerular filtration rate and APOE ε4 allele. Separate analysis were conducted for men and women. We applied causal mediation analysis to determine how plasma biomarkers mediate the association between PEF and incident dementia.

**Result:**

In models adjusted for covariates, men (β = 0.11 p = 0.09) and women ( = 0.06, p = 0.09) in SRP <10% show elevated GFAP levels compared to those in SRP ≥50%. Women in SRP <10% exhibit higher NfL levels (β = 0.13, p = 0.001) compared to those in SRP ≥50%. PEF showed no association with plasma levels of Aβ42/40 and p‐tau 181 in both sexes. In women, the association between lung function and incident dementia was mediated by plasma levels of NfL (proportion mediated = 20%, 95% CI = [1.2, 39], p = 0.04).

**Conclusion:**

Individuals with poor lung function exhibit elevated levels of NfL and GFAP in their blood, with discernible sex‐specific variations in the association. These findings suggest a potential connection between lung health and cognitive function in older women, and providing a basis for future hypotheses to explore the pathological mechanisms of AD/ADRD.

